# Assessing the impacts of short-course multidrug-resistant tuberculosis treatment in the Southeast Asia Region using a mathematical modeling approach

**DOI:** 10.1371/journal.pone.0248846

**Published:** 2021-03-26

**Authors:** Win Min Han, Wiriya Mahikul, Thomas Pouplin, Saranath Lawpoolsri, Lisa J. White, Wirichada Pan-Ngum

**Affiliations:** 1 Department of Tropical Hygiene, Faculty of Tropical Medicine, Mahidol University, Bangkok, Thailand; 2 HIV-NAT, Thai Red Cross AIDS Research Centre, Bangkok, Thailand; 3 Faculty of Medicine and Public Health, HRH Princess Chulabhorn College of Medical Science, Chulabhorn Royal Academy, Bangkok, Thailand; 4 Pharmacology Department, Mahidol-Oxford Tropical Medicine Research Unit (MORU), Bangkok, Thailand; 5 Centre for Tropical Medicine, Nuffield Department of Medicine, University of Oxford, Oxford, United Kingdom; 6 Li Ka Shing Centre for Health Information and Discovery, Nuffield Department of Medicine, Big Data Institute, University of Oxford, Oxford, United Kingdom; 7 Mathematical and Economics Modelling (MAEMOD) Research Group, Mahidol-Oxford Tropical Medicine Research Unit (MORU), Bangkok, Thailand; University of Illinois College of Veterinary Medicine, UNITED STATES

## Abstract

This study aimed to predict the impacts of shorter duration treatment regimens for multidrug-resistant tuberculosis (MDR-TB) on both MDR-TB percentage among new cases and overall MDR-TB cases in the WHO Southeast Asia Region. A deterministic compartmental model was constructed to describe both the transmission of TB and the MDR-TB situation in the Southeast Asia region. The population-level impacts of short-course treatment regimens were compared with the impacts of conventional regimens. Multi-way analysis was used to evaluate the impact by varying programmatic factors (eligibility for short-course MDR-TB treatment, treatment initiation, and drug susceptibility test (DST) coverage). The model predicted that overall TB incidence will be reduced from 246 (95% credible intervals (CrI), 221–275) per 100,000 population in 2020 to 239 (95% CrI, 215–267) per 100,000 population in 2035, with a modest reduction of 2.8% (95% CrI, 2.7%–2.9%). Despite the slight reduction in overall TB infections, the model predicted that the MDR-TB percentage among newly notified TB infections will remain steady, with 2.4% (95% CrI, 2.1–2.9) in 2020 and 2.5% (95% CrI, 2.3–3.1) in 2035, using conventional MDR-TB treatment. With the introduction of short-course regimens to treat MDR-TB, the development of resistance can be slowed by 38.6% (95% confidence intervals (CI), 35.9–41.3) reduction in MDR-TB case number, and 37.6% (95% CI, 34.9–40.3) reduction in MDR-TB percentage among new TB infections over the 30-year period compared with the baseline using the standard treatment regimen. The multi-way analysis showed eligibility for short-course treatment and treatment initiation greatly influenced the impacts of short-course treatment regimens on reductions in MDR-TB cases and percentage resistance among new infections. Policies which promote the expansion of short-course regimens and early MDR-TB treatment initiation should be considered along with other interventions to tackle antimicrobial resistance in the region.

## Introduction

Multidrug-resistant tuberculosis (MDR-TB) infections, including rifampicin-resistant TB (RR-TB), are a major issue for TB control and prevention programs and are considered to be a threat to global public health. There were an estimated 558,000 incident cases of MDR/RR-TB worldwide in 2017 and it is estimated that 3.5% of all cases of TB detected were MDR/RR-TB [1]. The World Health Organization (WHO) Southeast Asia Region has 6 of the 30 countries with the highest MDR-TB burden in the world and approximately 30% of all cases of MDR-TB worldwide were found among notified pulmonary TB cases in this region [2]. Although drug-resistant tuberculosis infections can be treated with more advanced and complex regimens of anti-tuberculosis drugs and even with recently approved drugs, such as delamanid and bedaquiline, under certain conditions [3, 4], the treatment for drug-resistant TB is still considered to be far from ideal due to the long duration of treatment and its toxicities.

With their superior safety and tolerability profiles, newly approved drugs for the treatment of drug-resistant TB infections have the potential to play an important role by being used in different combined treatment regimens. However, there is also a need for the development of new treatment regimens with a shorter duration, higher cure rate, improved tolerability, and lower cost. Recent studies from Niger, Cameroon, and Bangladesh have shown that new MDR-TB treatment regimens of 9 to 12 months have relapse-free cure rates ranging from 84% to 90% in patients without fluoroquinolone resistance [5–7]. In 2016, WHO recommended the short-course (9–11 months) MDR-TB treatment regimens under certain conditions, such as for patients without extrapulmonary TB and quinolone resistance or who were pregnant [8], although all-oral MDR-TB regimen was suggested to be the preferred option in the updated recommendation [9]. A multi-center randomized controlled trial (Standard Treatment Regimen of Anti-Tuberculosis Drugs for Patients with MDR-TB [STREAM]) is currently carrying out further evaluations of the safety and efficacy of these regimens [10]. Stage 1 results from the STREAM study showed that the short-course regimen was non-inferior to the conventional MDR-TB treatment regimen for 20 to 24 months (78.8% vs. 79.8%, *p* = 0.02 for noninferiority) [11].

Accessibility to better treatment regimens of shorter duration and fewer toxicities is recognized to be a key feature necessary to reduce TB cases and mortality and also to achieve the targets set by the United Nations’ Sustainable Development Goals (SDG) [12]. Previous modeling studies have also shown the likely benefits of short-course MDR-TB treatment when integrated with testing for drug resistance and eligibility for treatment [13, 14]. In addition, co-existing challenges, such as low MDR-TB detection rates, slow uptake of drug susceptibility testing (DST), eligibility for short-course treatment due to resistance to quinolone and other second-line anti-TB drugs, and delayed treatment initiation, mean the effect that the introduction of short-course treatment alone in the Southeast Asia Region would have on the burden of MDR-TB is unknown. Therefore, we constructed a transmission dynamics model for TB and MDR-TB, taking into account the different challenges faced in the Southeast Asia setting, to evaluate the likely population-level impacts of short-course MDR-TB treatment regimens under different programmatic scenarios.

## Methods

### Model structure

**Fig 1:**
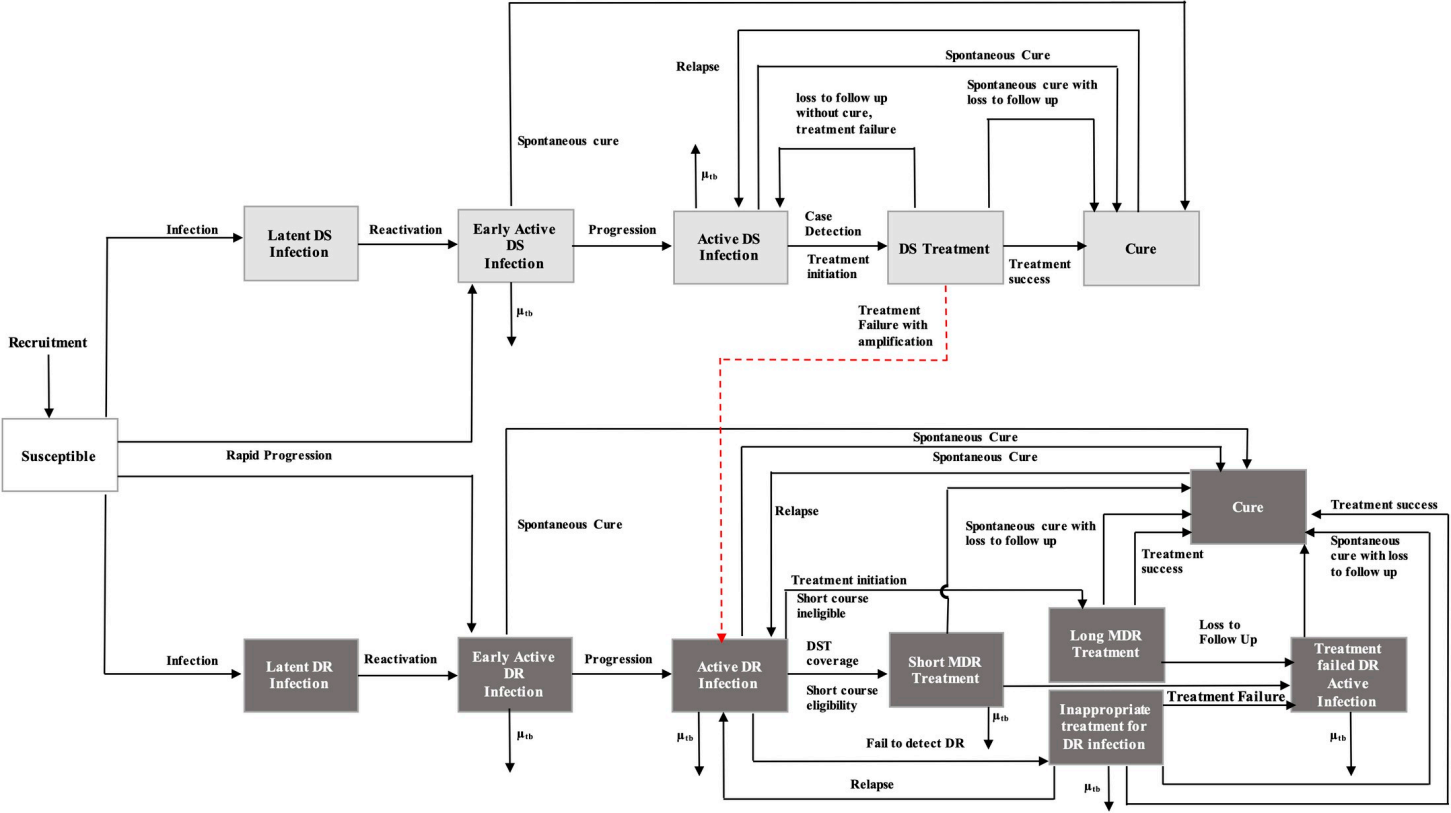
Model structure for drug-susceptible (DS)- and multidrug-resistant (MDR)-TB.

We built a compartmental deterministic model to consider pulmonary TB with two strains, drug-susceptible (DS) and multidrug-resistant (MDR), in a homogenous population (Fig 1). The model divided the population into compartments according to individuals’ TB disease state. Susceptible individuals could be infected with either the DS or the MDR strain, depending on their exposure. The disease states continued with either rapid progression to early active infection or latent infection compartments. While latently infected individuals could reactivate to active infections at a given rate, early active infections were able to progress to clinically active states; these active infections could then be detected and commence anti-tuberculosis treatment. The rates of treatment initiation were equal for both strains, but the case detection rates for MDR-TB at initial infection were lower, to reflect the limited availability of DST in some settings in the Southeast Asia Region [15–17].

Following the initiation of treatment, outcomes were further classified according to the probabilities of cure, treatment failure with or without amplification (acquired drug resistance during treatment), loss to follow up, and with or without spontaneous cure. Individuals who acquired resistance during treatment entered the MDR-TB compartment (red line in the model structure, shown in Fig 1). If those individuals were eligible (without resistance to a shorter MDR-TB regimen), they were treated with short-course MDR-TB treatment. MDR-TB infections that were not diagnosed due to limited availability of DST were inappropriately treated as DS-TB infections. It was assumed that MDR-TB exhibited reduced transmission efficiency compared with DS-TB, as was the case in previous TB models [18]. A list of parameters used in the model is shown in Table 1. We assumed that inappropriate treatment yielded less favorable outcomes compared with outcomes following either short-course or conventional treatment. Relapses could occur among the cured populations following treatment for either DS- or MDR-TB infections. The model structure, including different diseases states and the ordinary differential equations (ODEs) and parameters used, are discussed in the S1 Appendix.

**Table 1:** Parameter table.

	DS-TB		MDR-TB					
Parameters	Value	Range	Value		Range	Distribution		Source
Proportion of treatment success	0.83		0.78^‡^			Logit-normal		[1, 38, 39]
			0.52^¶^					
Proportion of lost to follow up	0.05		0.08^‡^			Logit-normal		[1]
			0.13^¶^					
Proportion of treatment failure	0.10		0.14^‡^			Logit-normal		[19, 40, 41]
			0.35^¶^					
Proportion of relapse after treatment	0.05	0.03–0.10	0.024		0.01–0.33	Logit-normal		[42–46]
Treatment initiation rate (per year)	1.3	0.5–2	0.7		0.5–1	Log-normal		[47, 48]
Proportion of infectious individuals with early active TB	0.48	0–1				Log-normal		Assumption
Proportion of infectious individuals receiving ineffective treatment	0.24	0–0.50						
Rate of progression from early active to fully active TB	1.2	0.7–1.8				Log-normal		[49]
Spontaneous cure rate	0.13	0.08–0.21				Log-normal		[50]
Proportion of acquired drug resistance	1-δ_s_-dff_s_-ε_s_					Logit-normal		
Duration of treatment for DS-TB (years)	0.5	0.4–0.75				Log-normal		[1]
Duration of treatment for MDR-TB (years)	2	1.75–2.2				Log-normal		[1]
Average duration to relapse (years)	1.7	0.9–2.5				Log-normal		[51]
Proportion receiving DST	0.5	0.4–0.8				Logit-normal		Assumption
Reduced probability of spontaneous recovery rate for those on inappropriate DR treatment	0.4	0.1–1				Uniform		Assumption
Mortality rate for TB without treatment	0.5	0.1–2				Log-normal		[52]
Mortality rate on TB treatment	0.09	0.05–0.14				Log-normal		[53]
**Model fitting**								
**Parameters**	Median	95% credible intervals (CrI)		Distribution			Supporting references	
Proportion of rapid progression to active TB *(upsilon)*	0.04	0.03–0.25		See posterior distribution (S2 Fig in S1 Appendix)			[18, 54]	
Transmission coefficient of TB *(beta_s)*	9.8	5–20		See posterior distribution (S2 Fig in S1 Appendix)			[24]	
Relative transmissibility of MDR-TB compared with DS-TB *(fitness)*	0.5	0–1		See posterior distribution (S2 Fig in S1 Appendix)			[25]	
Reactivation rate from latent TB to active disease per year *(psi)*	0.001	0.0005–0.002		See posterior distribution (S2 Fig in S1 Appendix)			[26, 27]	

^‡^Parameter values for conventional MDR-TB treatment.

^¶^Parameter values for short-course MDR-TB treatment.

### Model simulations

An initial run-in period of 65 years was executed, starting from the year 1955 and running to 2020. Treatment interventions were added to the model in the year 1955 and hence allowed the amplification of drug-resistant strains through the treatment of drug-sensitive strains. The incidence of MDR-TB includes a combination of reactivation, rapid progression to MDR-TB, and amplification. Resistant infections may arise from the product of the force of infection (FOI), be derived from resistant and susceptible infections (becoming latent and then active later), or they may be *de novo* resistance, arising from amplification.

### Model validation and projection

Where available, estimates from published literature were used for the model parameter values. The remaining model parameters were estimated through fitting the model to the WHO Southeast Asia Region TB incidence data from 1990 to 2017 of all TB cases per 100,000 population and percentage of MDR-TB among newly notified cases (S1 Table in S1 Appendix), via the Markov Chain Monte Carlo (MCMC) method, implemented in the Bayesian Tools R package [19]. The parameters estimated by the fitting were the proportion of rapid progression to active disease, the transmissibility of MDR-TB infections, relative fitness of drug-resistant TB compared with the transmissibility of the DS strain and rate of endogenous reactivation from latent to early active TB. The model was further used to project TB incidence, estimated number of cases of MDR-TB, and the percentage of new MDR-TB cases among newly notified cases up to year 2035, sampling the parameters from the posterior chains. The model was constructed and computed using R programming software version 3.2.3 (R Core Team, Vienna, Austria) [20], the package for differential equations [21], and the Bayesian Tools package for model validation [22].

### Treatment intervention scenarios

Once the model had been validated, it was used to assess the impacts of using the standard regimen and comparing this with the shorter MDR-TB treatment. Data from available cohort studies suggested that shorter durations of treatment for MDR-TB were not inferior to the standard regimen used within current TB programs [6, 7]. According to recent recommendations from WHO on the use of short-course treatment regimens for MDR-TB, it was advised not to use such treatments for those who have extrapulmonary TB or quinolone resistance [23]. Based on the evidence reported from clinical trials [6, 7], a shorter regimen is superior to the standard treatment in the following ways:

1. Treatment completion rates with shorter regimens are higher than for the standard regimen,
2. Lower loss to follow-up rates are seen with shorter treatment regimens, and
3. The probabilities of cure rates with the shorter regimens are higher than with the standard regimen.

However, the benefit of using the shorter regimen will also depend greatly on programmatic factors. The successful treatment associated with the MDR-shorter course regimen could be determined by three factors: i) eligibility–the proportion of patients with MDR-TB eligible for the short-course MDR-TB regimen, ii) coverage of DST–the proportion of patients with MDR-TB receiving DST, and iii) treatment initiation rate. Multi-way analysis was performed to assess the associations among these factors and the success of short-course MDR-TB treatment regimens on MDR-TB. Each of these factors was categorized into three levels, varying over a range of values, as shown in Table 2. The model was run to 2035 and the results in terms of reductions in MDR-TB cases and MDR-TB percentage when compared with the baseline scenario were recorded.

**Table 2:** Programmatic scenarios used in the model projections along with the evaluation of short-course MDR-TB regimens.

Programmatic scenario	Baseline value	Ranges used in the programmatic scenario	Source
Eligibility for a short-course regimen (%)	0	50, 80, 100	[1]
DST coverage (%)	50	80, 90, 100	[1]
Time to diagnosis and treatment initiation (months)	12	1, 5, 10	[1]

## Results

### Model validation and projections

**Fig 2:**
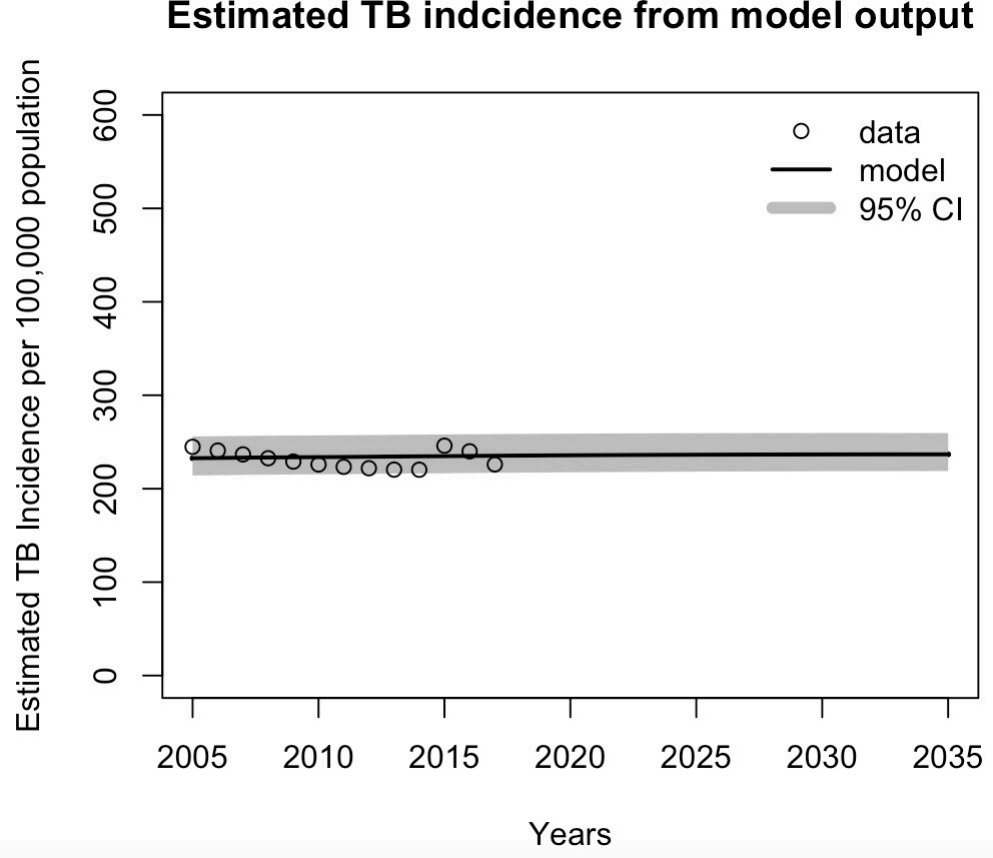
Model projection of estimated TB incidence per 100,000 population, with credible intervals. The black line shows the model results of estimated TB incidence, and the dots represent WHO data for estimated TB incidence.

**Fig 3:**
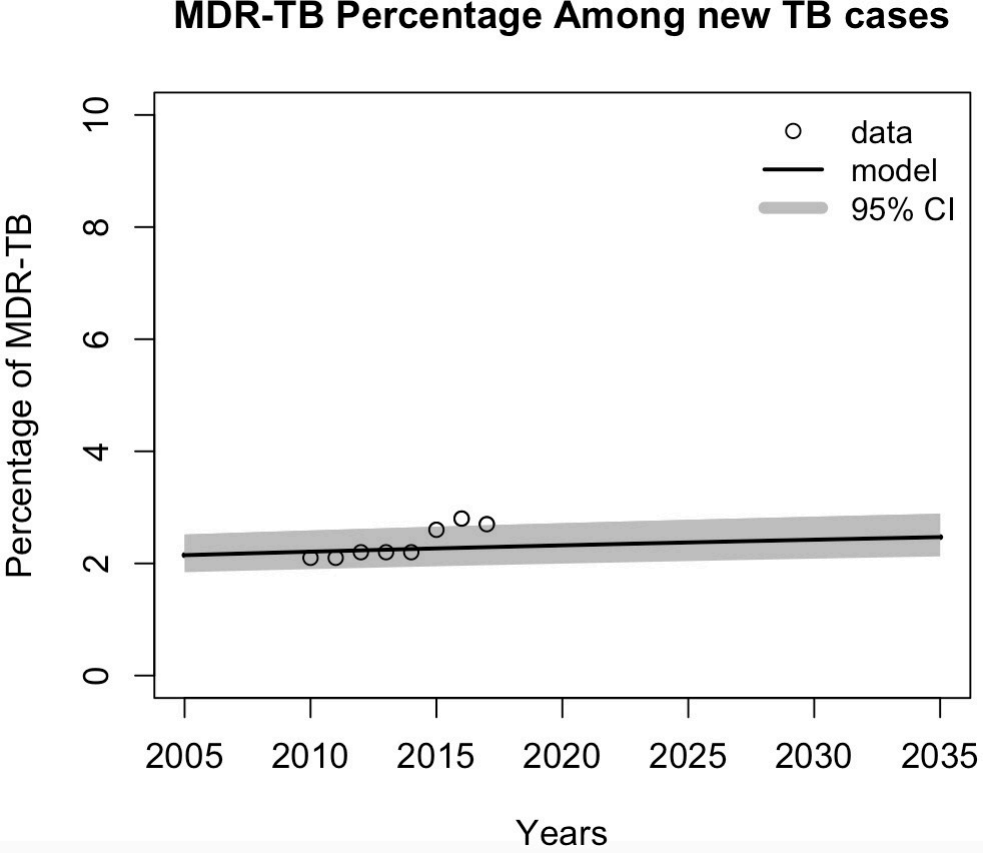
Model projection of estimated MDR-TB percentage among new TB cases. The black line shows the model results of percentage of MDR-TB cases, and the dots represent WHO data for the estimated proportion of MDR-TB among new tuberculosis infections.

**Fig 4:**
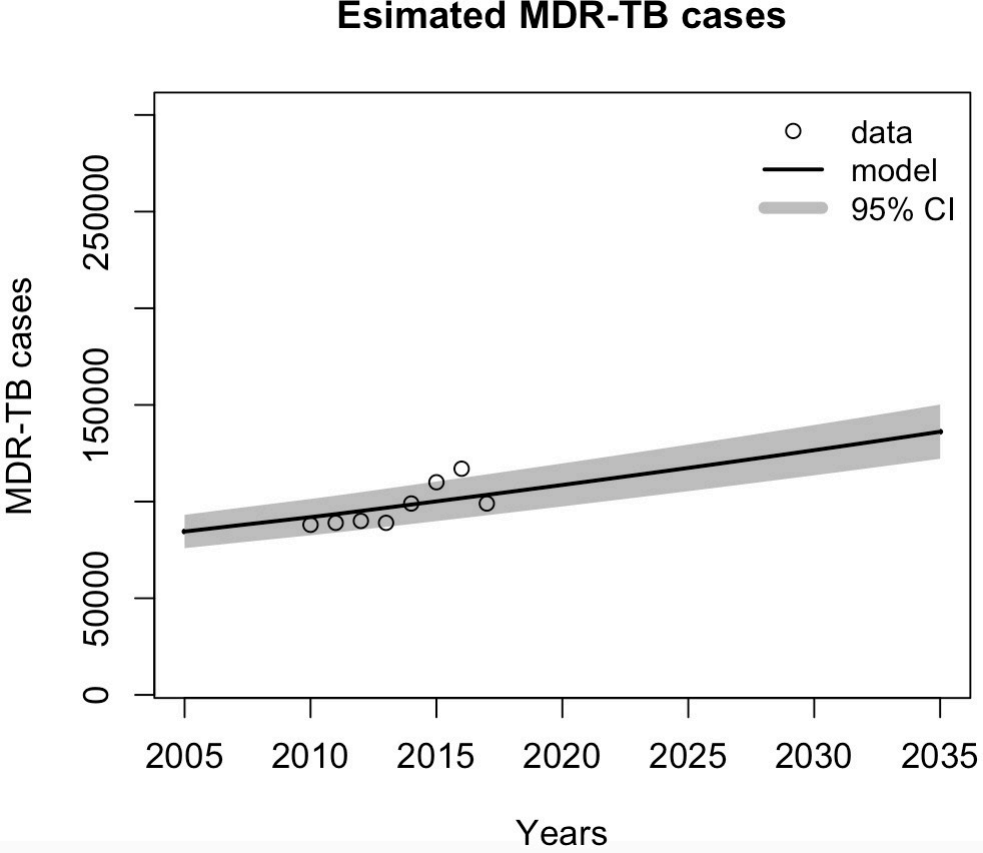
Model projection of estimated numbers of MDR-TB cases. The black line shows the model validation results of numbers of MDR-TB cases, and the dots represent WHO data for the numbers of estimated MDR-TB cases.

Given the current strategy, the model predicted that overall TB incidence would decline over time (Fig 2), although the percentage of MDR-TB cases among new cases (Fig 3) and overall MDR-TB cases (Fig 4) would increase. The projections of TB incidence and MDR-TB percentage among newly notified cases are shown in Figs 2 and 3, with 95% credible intervals (CrI). Overall, the model projected that TB incidence will decrease, from 246 (95% CrI, 221–275) per 100,000 population in 2020 to 239 (95% CrI, 215–267) per 100,000 population in 2035, a modest reduction of 2.8% (95% CrI, 2.7%–2.9%). The model predicted a relative increase in total MDR-TB cases of 4.2% (95% CrI, 3.9–9.5) and MDR-TB percentage among TB infections of 23.9% (95% CrI, 23.8–24.1) in 2035, compared to 2020. These model simulation results were then used as the baseline for the subsequent analysis. In the model validation process, we estimated four parameters and reported the median values and posterior distribution. For each parameter, the value fell into the ranges reported from some previous studies i.e. proportion of rapid progression to active TB [18], transmission coefficient [24], relative transmissibility of MDR-TB compared with DS-TB [25], and reactivation rate from latent TB to active disease per year [26, 27] (see Table 1). Although these parameter values used in the fitting and prediction process are based on the incidence and resistance, the variation in the parameter values is included to reflect the uncertainty in the predictions, and the posterior distributions represent sets of collinear parameters that reproduce the observed data (S2 and S3 Figs in S1 Appendix). Further studies and more appropriate data are required to refine these parameters.

### Impacts of shorter course MDR-TB treatments under different conditions

The baseline scenario assumed no short-course treatment available, DST coverage of 50% and treatment initiation is one year on average. The estimated number of MDR-TB cases in 2035 was around 132,000 in total (Fig 4), while the percentage of resistance was 2.54% in the same year (Fig 3). We modeled the impacts of using shorter course treatment regimens under different conditions that influenced successful treatment outcomes. These conditions were based on combinations of different levels of treatment eligibility, DST coverage, and treatment initiation. The model was used to predict the numbers and percentages of MDR-TB from 2020 to 2035 for the Southeast Asia Region. The impacts of the short-course treatment regimen were evaluated by comparing the estimated number of individuals with MDR-TB infections and the estimated percentage of MDR-TB cases among new TB cases with those of the baseline scenario, where no short-course regimens were introduced.

**Fig 5:**
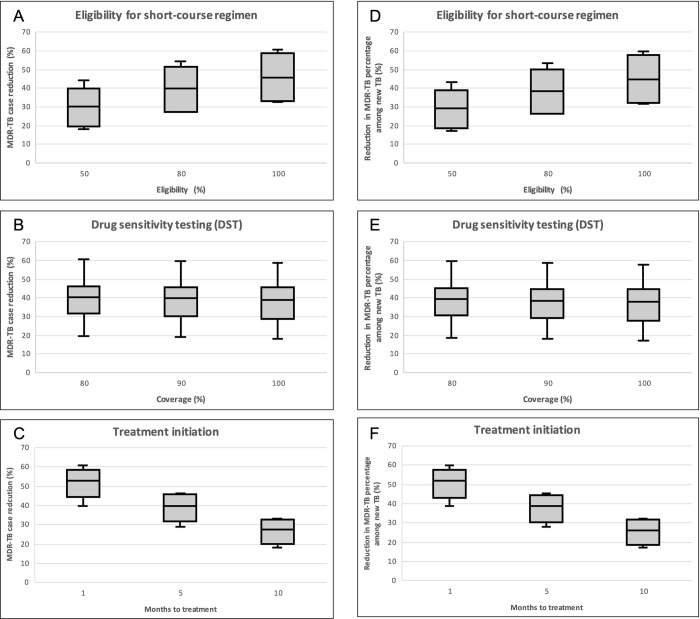
Multi-way analysis focusing on three factors (eligibility, DST and treatment initiation) influencing the impacts from the short-course regimens (Table 2). The outcomes in term overall MDR-TB cases (A-C) and MDR-TB percentage among new cases (D-F) from 81 combinations were simulated and compared to the baseline (using the standard regimen). Fig 3 categorized the outcomes by eligibility (A & D), DST (B & E), and treatment initiation (C & F)., shown in boxplots.

The model’s predictions showed that the effectiveness of short-course regimens is significantly dependent on treatment eligibility and the rate of treatment initiation, while DST coverage had little influence on the outcomes (Fig 5). Given some variations in the proportion of DST and treatment initiation rate, Fig 3A and 3D show that the higher the proportion of patients who are eligible to receive the short-course treatment, the greater the effect in controlling MDR-TB cases and resistance percentage. The estimated percentage of reduction in MDR-TB cases and MDR-TB percentage among newly notified TB cases when the eligibility is 50% or more was approximately 38.6% (95% confidence intervals (CI), 35.9–41.3) and 37.6% (95% CI, 34.9–40.3), respectively, compared to with the baseline scenario. The model is also sensitive to the treatment initiation, the sooner the patients received their treatment, the higher the impact of starting a short-course regimen. Ideally, patients should begin treatment within one month of active infection being diagnosed; this would result in a reduction in MDR-TB cases when using the short-course regimen of 51.3% (95% CI, 48.4–54.1) and a 50.3% (95% CI, 47.4–53.1) reduction in the proportion of MDR-TB cases among new infections.

## Discussion

The population-level impacts of short-course treatment regimens for MDR-TB in the Southeast Asia Region are uncertain. However, our study has shown that new MDR-TB treatments of shorter duration and their associated effective treatment responses could be beneficial for the prevention and control of MDR-TB if they were to be combined with improvements in other key programmatic factors, such as increased rates of treatment initiation and with a reasonable proportion of patients eligible for such regimens. Our model also showed that MDR-TB treatment interventions were predicted to have very little effect on changing the overall incidence of TB infections due to the low prevalence of MDR-TB. However, significant results were predicted for MDR-TB treatment outcomes, showing a reduction in the percentage of MDR-TB among new TB cases and an increase in the number of new MDR-TB cases averted.

Due to the urgent need for new MDR-TB treatments to control the current epidemic in the Southeast Asia Region, we developed a mathematical model to predict the outcomes of implementing new treatment regimens in the region. Such treatment programs are complex, and many issues must be addressed to achieve the successful control of MDR-TB. For example, in countries such as India and China, the transmission of drug resistance is of major concern because of the influence of population density. Recent studies, including both whole-genome sequencing [28] and mathematical modeling projections [18], have provided evidence to support the hypothesis that the spread of drug resistance contributes a much greater percentage of MDR-TB than the *de novo* development of resistance from the amplification of drug-sensitive TB cases.

Our model projected that the optimal strategy would include having a greater proportion of patients who are eligible for short-course treatment regimens. This approach was predicted to yield the greatest impact on reducing the percentage of MDR-TB cases among new TB cases and on the estimated number of MDR-TB cases, compared with other interventions under different scenarios. The model also predicted the likely beneficial impacts of different interventions with varying proportions of the coverage of DST and treatment initiation rates. Previous modeling studies have also suggested the potential benefits of using short-course regimens on the population-level impact of the MDR-TB burden; however, the efficacies of the drugs used will depend on the coverage of treatment accessibility and DST availability [13, 25]. Recent studies have shown that the introduction of rapid molecular DSTs at TB diagnosis for both first- and second-line treatment improved treatment and survival outcomes in patients with MDR-TB who were receiving treatment [29, 30].

It is notable that these predicted impacts would not be applicable if other factors were to influence or limit treatment initiation, DST coverage, or the use of the correct treatment regimen. Our model used parameters for treatment outcomes of a new, shorter treatment regimen based on cohorts in settings with low or no fluoroquinolone resistance [6, 7]; treatment outcomes from trials such as these may not be the same as those from actual programmatic outcomes. For example, the loss to follow up rate tends to be higher in actual settings than in trials. In addition, the duration of and compliance with an MDR-TB treatment regimen in controlled trials tend to have better outcomes than in actual programmatic settings. However, one would expect that treatments of shorter duration would be beneficial for patients, healthcare facilities, and TB programs due to the reduction in clinic visits and decreased financial and time burdens for patients, which could be a potential opportunity for decreasing the loss to follow-up.

Despite these caveats, short courses for MDR-TB treatment–regimens recently recommended by WHO in 2016 –could be considered according to their clinical effectiveness and future cost-effectiveness in a given setting. Such short courses of treatment are encouraging and hopeful, not only because of their impacts on transmission but also because of their cost-effectiveness and their benefits both to infected individuals and to national health programs. Nonetheless, optimal guidance for the use of newer treatment regimens is urgently needed, as alarming recent reports have suggested the emergence of acquired resistance to newer anti-TB drugs (such as delamanid, with mutations in the *fbiA* and *fgd1* genes) due to inadequate MDR-TB and extensively drug-resistant TB (XDR-TB) treatment regimens for individuals who already have infections resistant to quinolone and other second-line anti-TB drugs [31, 32].

Our model has several limitations. Due to the relatedness of some parameters considered in the model and the HIV situation in some settings, the model’s predictions could be highly influenced by these factors. For example, in settings where there is a high prevalence of HIV, a parameter such as the reactivation rate from latent to active TB infection may be accelerated, the chance of acquiring MDR-TB may be greater due to weaker immune responses resulting from HIV infection, there may be unforeseen interactions with antiretroviral therapies [33], and the risk of mortality may be higher. Any or all of these variations could influence our primary analysis. Our study focused on the WHO Southeast Asia Region as a whole, and so our results and findings may not be relevant to MDR-TB transmission in other settings, for example in China, where TB incidence is declining rapidly but there is an increasing burden of MDR-TB, or in South Africa, where loss to follow-up during treatment is one of the major challenges faced in controlling the TB burden. A further limitation of our study is that we did not include resistance to individual drugs or mono-resistant transmission, for example isoniazid resistant-only cases. We also did not evaluate the impact of treatment for latent MDR-TB, which could be a potential intervention for reducing the MDR-TB burden in the region. A recent modeling study also evaluated the burden of latent MDR-TB in different regions, including countries in Southeast Asia [34], and there is currently an ongoing clinical trial (ClinicalTrials.gov: NCT03568383) to evaluate the efficacy of treatment for latent MDR-TB among the household contacts of index cases. Although we estimated the fitness cost of MDR-TB strains exhibiting reduced transmissibility compared with the transmissibility of DS-TB strains, this estimate is still uncertain and subject to further analyses by other sets of data. In the absence of the fitness cost, the transmission potential of MDR-TB may depend on infectiousness, frequency of contacts, or duration of disease, all of which may influence the epidemic trends [35, 36]. A recent large prospective study showed that household contacts exposed to MDR-TB had a higher risk of becoming infected than those exposed to DS-TB, which suggests there are minimal transmissibility deficits between DS-TB and MDR-TB strains [37].

## Conclusions

Although our study predicted that the incidence of TB will decrease over the next 15 years, the incidence of MDR-TB is projected to continue increasing. Our model predicted that short-course treatments for MDR-TB could reduce the MDR-TB burden, especially if combined with improvements in other key programmatic factors. It also predicted that combining short-course regimens with other interventions, such as increasing treatment eligibility, coverage of DST, and treatment initiation, could potentially provide greater benefits in reducing the burden of MDR-TB. More importantly, comprehensive cost-effectiveness analyses of the newer MDR-TB or XDR-TB treatment regimens and more clinical trials of these short-course regimens with all oral medications are needed. Also, further studies are warranted to better understand the efficiency of these treatments, optimal treatment allocations, price negotiations, and quantitative measures of both the direct benefits of these regimens to individuals infected with MDR-TB and indirect benefits at the population level.

## Supporting information

S1 Appendix (DOCX)
