# Supplementary material for: Assessing the impacts of short-course multidrug-resistant tuberculosis treatment in the Southeast Asia Region using a mathematical modeling approach
Source: PLoS One. 2021 Mar 26;16(3):e0248846. doi: 10.1371/journal.pone.0248846 (PMC7997007; doi:10.1371/journal.pone.0248846)
Supplement: S1 Appendix — (DOCX) [file pone.0248846.s001.docx]

**S1 Appendix**

1. **Model Construction**

A deterministic compartmental model of tuberculosis (TB) transmission was constructed using ordinary differential equations. A layer of resistance status, i.e., drug-sensitive (DS) versus multi-drug resistance (MDR), was built on top of the model to measure the spread of resistance during transmission. The various compartments used are described below (please also see the model structure in **Fig 1** in the main text).

### **1.1 Susceptible (S) compartment**

To reduce the complexity of the model, BCG vaccination, population migration, and age groups were not included in the model. Although BCG vaccination is efficacious for preventing TB in the years following immunization, this immunity can be overwhelmed by frequent exposure to infection, especially in settings with a high burden of TB.

**1.2 Latent infection (L_s_ and L_r_) compartments and active infection (I_s_ and I_r_) compartments**

Due to the different rates of progression to active TB infection and the prolonged period of latent infections in the natural history of TB infection, it was practical to set different rates for the development of TB infection in the model. Almost 10% of individuals with a latent TB infection, as confirmed by a positive interferon-gamma releasing assay, progressed to active TB disease within two years [1]. “Normal” and “fast” pathways, with a proportion moving from susceptible to latent infection, then reactivated to active infection, and a proportion moving from susceptible and directly progressing to active TB infection, were included in this model as compartments **I_s_** and **I_r_**.

### **1.3 Treatment (T_s_ and T_r_) compartments**

Not all individuals with active TB disease are diagnosed and started on the appropriate treatment. Previous models with pre-diagnosis delays and delays to treatment following a diagnosis have been explored [2, 3]. The sensitivity of a diagnostic test, multiplied by the rate of progression from susceptible to active infection, can be integrated to quantify the under-treatment of TB due to errors in diagnosis. However, by assuming active TB cases detected were diagnosed and appropriately treated, we incorporated all stages, from active infection to the initiation of anti-TB treatment, in the same compartment as the treatment initiation rate (ω).

### **1.4 Cured (R_s_ and R_r_) compartments**

The cure or recovered state of tuberculosis infection includes the completion of treatment and meets the definition of cure; however, following completion of their treatment not all individuals achieve the cured state. There is also a proportion of individuals with active TB infections who experience spontaneous or self-cure without commencing anti-TB treatment, or who are lost to follow up after having treatment for a certain period of time in a treatment program.

Relapse or reinfection following treatment has been observed, especially in immunocompromised patients, and has been confirmed using DNA fingerprinting techniques [4]. A recurrent episode of active TB disease could be due to either subsequent infection with a new strain of TB or relapse with the same strain, and various results for these rates were observed in a previous study [5]. The current model allowed patients to move from a cured state to a compartment of recurrent active infection that has previously been treated with anti-TB drugs.

### **1.5 Recurrent active infection (I_2s_ and I_2r_) compartments**

There are several pathways to enter the recurrent active infection compartments in our model: individuals who completed treatment but did not meet the state of being cured, individuals who were lost to follow up or defaulted from the treatment program, and those who failed to respond to anti-TB treatment. As with first time active infection, individuals who have previously been treated for another episode of TB disease may be cured spontaneously but at a lower rate.

### **1.7 Risk of acquiring drug resistance**

Without amplification, patients who defaulted on their treatment returned to the infected compartment. In some previous models, amplification rates were considered to be proportional to the rates of treatment of drug-sensitive strains [6, 7]. In this model, amplification was assumed to occur in treatment failure cases, moving some drug-sensitive infections that had treatment episodes to the resistant compartments. For model simplicity, we focused on MDR-TB transmission; amplification from MDR-TB to extensively drug-resistant TB (XDR-TB) was not included in this model.

1. **Compartments**

$\boldsymbol{S}$= the number of susceptible individuals for drug-sensitive and drug-resistant TB

$\boldsymbol{L}_{\boldsymbol{s}}\boldsymbol{,}\boldsymbol{L}_{\boldsymbol{r}}$ = the number of drug-sensitive/resistant individuals with latent TB infection

**EIs** = the number of early active infections with drug-sensitive TB

$\boldsymbol{I}_{\boldsymbol{s}}\boldsymbol{,}\boldsymbol{I}_{\boldsymbol{r}}$= the number of fully active infections with drug-sensitive/resistant TB

**Ts** = the number of individuals receiving first-time anti-TB treatment for drug-sensitive TB

**Rs** = the number of individuals who recovered following first-time anti-TB treatment of drug-sensitive TB

**Lr** = the number of individuals with latent MDR-TB infection

**EIr** = the number of early active MDR-TB infections

**Ir** = the number of initial active MDR-TB infections

$\boldsymbol{I}_{\boldsymbol{r}}^{\boldsymbol{bad}}$= the number of active MDR-TB infections on inappropriate treatment

$\boldsymbol{T}_{\boldsymbol{r}}^{\boldsymbol{short}}\boldsymbol{,}\boldsymbol{T}_{\boldsymbol{r}}^{\boldsymbol{long}}$ = the number of individuals receiving first-time anti-TB treatment for MDR-TB (short course and long course)

**Rr** = the number of individuals who recovered from first-time anti-TB treatment for MDR-TB

$\boldsymbol{I}_{\boldsymbol{r}}^{\boldsymbol{fail}}$ = the number of individuals who failed MDR-TB treatment

1. **Parameter samples and ranges**
2. **Probability of rapid progression to active TB:** A fraction of individuals who are fully susceptible to either DS- or MDR-TB progressed to early active TB after infection. We sample the range from the previous literature [8]. The estimated value of probability of rapid progression to TB disease after infection was consistent with previous study [9].
3. **Transmission coefficient of TB**: This value was calibrated based on WHO TB incidence data [10], which were used for the model fitting and forecasting purposes. The estimated range was also verified for consistency using independent estimates of effective contact rates from the literature [11].
4. **Relative transmissibility of MDR-TB compared with DS-TB** : We sampled a full range of possible values (0-100%) for the relative transmissibility of MDR-TB compared to DS-TB. The estimated value was also consistent with the previous modelling works [12].
5. **Reactivation rate from latent TB to active disease**: The reactivation rate depends on the degree of reinfection in a particular setting. For example, low- and middle-income settings with a high level of reinfections have a higher reactivation rate compared with the reactivation rate in high-income settings with low reinfection rates. The early years of an infection have the highest risk for progression to active disease. In contrast, the reactivation rate is much lower after this initial high-risk period [13]. We used the sample from the literature of reactivation rate in a systematic review [14] and the value of the rate of 3-5 years after infection [15].
6. **Differential equations**

**Susceptible (uninfected) state:** Individuals in the initial phase could be susceptible to either drug-sensitive or -resistant strains of TB. The rate of change in susceptible state was calculated as a balance between the force of infection, rapid progression to an active infection, and the natural mortality rate (μ).

$$\frac{dS}{dt}= \pi. P- \lambda_{s}.S- \lambda_{r}.S- \mu.S$$

All equations with a lowercase “s” refer to flows describing “drug-sensitive” states, whereas a lowercase “r” represents a “drug-resistant” state.

**Latently infected state:** The fraction entering the latent infection state is determined by the fraction of individuals who do not rapidly progress to the active disease state (1-$\upsilon$) with the effective contact rate of TB infection. The rate of change from latent infection state was calculated as a balance of the reactivation ($\psi$) to an early active infection or natural mortality (μ).

$$\frac{dL_{s}}{dt}= \lambda_{s}.\left( 1- \upsilon\right).S-\psi.L_{s}-\mu.L_{s}$$

$$\frac{dL_{r}}{dt}= \lambda_{r}.\left( 1- \upsilon\right).S-\psi.L_{r}-\mu.L_{r}$$

**Early active disease state:** This compartment gains individuals from rapid progression following an initial infection from the susceptible compartment and endogenous reactivation in latently infected individuals. The rate of change from this state was a balance of the rate of progression into fully active infection (alpha, α_s_), spontaneous recovery (phi, φ), or natural mortality (μ).

$$\frac{dEI_{s}}{dt}= \lambda_{s}. \upsilon.S+ \psi.L_{s}- \alpha.EI_{s}- \phi.EI_{S}- \mu_{tb}.EI_{s}- \mu.EI_{s}$$

$$\frac{dEI_{r}}{dt}= \lambda_{r}. \upsilon.S+ \psi.L_{r}- \alpha.EI_{r}- \phi.EI_{r}-\mu_{tb}. EI_{r}- \mu.EI_{r}$$

**Active disease state:** This develops via the progression from early active disease, with losses to the treatment compartment according to treatment initiation rate (ω), as well as to the recovered compartment with spontaneous resolution (phi, φ). If individuals have previously been treated with anti-TB chemotherapy, regardless of treatment frequency, failure, or success, and have had no drug resistance detected, they will stay in the previously treated active DS state until the next treatment commences. Additionally, a balance of relapses of infections in cases who recovered from either initial treatment or retreatment was calculated as the rate of change for this state.

*Drug-sensitive tuberculosis infections*

The active state of the DS-TB infections was describe as:

$$\frac{dI_{s}}{dt}= \alpha.EI_{s}-\phi.I_{s}+\omega_{s}. I_{s}+\frac{1}{treat_{s}^{duration}}.dff_{s}.T_{s}+\frac{1}{treat_{s}^{duration}}.\varepsilon_{s}.T_{s}+ \frac{1}{relapse_{duration}}. \kappa_{s}.R_{s} -\mu_{tb}.I_{s}-\mu.I_{s}$$

*Drug-resistant tuberculosis infections*

Those whose infection developed from the acquisition of resistance during treatment (initial or re-treated) or who progressed from early active MDR-TB disease (EI_r_), will be in active MDR-TB infection, until they have been diagnosed, spontaneously recovered, or died (either through natural or TB-related causes).

$$\frac{dI_{r}}{dt}= \alpha.EI_{r}+ \iota_{s}.\frac{1}{treat_{s}^{duration}}T_{s}+\frac{1}{treat_{s}^{duration}} .dff.T_{r}^{bad}+\frac{1}{treat_{s}^{duration}} .\varepsilon_{r}^{bad}.T_{r}^{bad}+\frac{1}{relapse_{duration}}. \kappa_{s}.R_{r}-\omega_{r}. I_{r}.- \phi.I_{r}- \mu_{tb}.I_{r}-\mu.I_{r}$$

*Drug-resistant infections due to improper treatment*

For the active MDR-TB infection state after treatment failure, those who are identified as MDR-TB and have failed due to either treatment failure or loss to follow up, from either short-course or standard MDR-TB treatment, will be included. Those who have failed previous MDR-TB treatment will be not re-treated and will remain until their infection resolves spontaneously or they die.

$$\frac{dI_{r}^{failed}}{dt}= \frac{1}{treat_{short}^{duration}}.\left( dff_{r}^{short}+\varepsilon_{r}^{short} \right).T_{r}^{short}+\frac{1}{treat_{long}^{duration}}. \left( dff_{r}^{long}+\varepsilon_{r}^{long} \right).T_{r}^{long}-\left( \phi+\mu_{tb}+\mu\right).I_{r}^{failed}$$

**Treatment state:** Individuals with active disease who are diagnosed as DS-TB will be treated at the rate of treatment initiation (ω). Patients who have started treatment will exit the treatment compartment with loss to follow up (dff), treatment success (delta, δ), or spontaneous recovery (phi, φ), to the cured compartment, and acquired drug resistance (iota, ι) with treatment failure rate (epsilon, ε) during the treatment phase. Those whose treatment fails and they do not acquire drug resistance will go to the compartment for previously-treated active disease. This compartment is for the treatment of recurrent active TB infections that have previously been treated. Losses from the compartment include those lost to follow up, success from treatment (bacteriological response to retreatment), spontaneous cure, and those who acquire drug resistance during treatment (it is assumed that the rate of amplification for this treatment state is higher than that with initial treatment).

$$\frac{dT_{s}}{dt}= \omega_{s}. I_{s}-\frac{1}{treat_{s}^{duration}} .\delta_{s}.T_{s}-\frac{1}{treat_{s}^{duration}} . dff_{s}.T_{s}- \frac{1}{treat_{s}^{duration}}.\varepsilon_{s}. T_{s}-\iota_{s}\frac{1}{treat_{s}^{duration}}.T_{s}-\phi.T_{s}-\mu_{tb}.T_{s}$$

Individuals who are eligible for a short course of MDR-TB treatment (1-g) will be initiated with the treatment initiation rate (ω_r_). In this state, there will be patients who respond to the treatment, who experience treatment failure, are lost to follow up, or spontaneously recover. Those who are not eligible for shorter treatment (g), with resistance to either quinolone or other second-line drugs, will be started on standard MDR-TB treatment.

$$\frac{dT_{r}^{long}}{dt}=b.\omega_{r}. I_{r}-\frac{1}{treat_{long}^{duration}}. \left( \delta_{r}^{long}+dff_{r}^{long}+\varepsilon_{r}^{long} \right).T_{r}^{long}-\phi.T_{r}^{long}-\mu_{tb}. T_{r}^{long}$$

$$\frac{dT_{r}^{short}}{dt}={b.\omega}_{r}.\left( 1-g \right). I_{r}-\frac{1}{treat_{short}^{duration}}.\left( \delta_{r}^{short}+dff_{r}^{short}+\varepsilon_{r}^{short} \right).T_{r}^{short}-\phi.T_{r}^{short}-\mu_{tb}. T_{r}^{short}$$

An ineffective treatment state in patients who have MDR-TB infection but are not diagnosed as having a resistant strain due to a lack of DST and are given DS-TB treatment with a mortality rate ($\mu_{tb}$).

$$\frac{dT_{r}^{bad}}{dt}=\left( 1-b \right).\omega_{r}.I_{r}- \frac{1}{treat_{s}^{duration}}.\delta_{bad}. T_{r}^{bad}- \frac{1}{treat_{s}^{duration}}.\varepsilon_{r}^{bad}.T_{r}^{bad}- \frac{1}{treat_{s}^{duration}}.dff.T_{r}^{bad}-i_{r}^{bad}.\phi.T_{r}^{bad}-\mu_{tb}.T_{r}^{bad}- \mu.T_{r}^{bad}$$

**Recovery state:** Patients who have been successfully treated or defaulted their treatment but whose infection resolved spontaneously and individuals with early active disease or active disease who had spontaneous resolution without any treatment, will go into the recovered state. The patients in this state remain noninfectious until they relapse or are reinfected with either DS or MDR strains.

$$\frac{dR_{s}}{dt}= \phi.I_{s}+ \phi. EI_{s}+\frac{1}{treat_{s}^{duration}}. \delta_{s}.T_{s}+\phi.T_{s}-\frac{1}{relapse_{duration}}. \kappa_{s}.R_{s} - \mu.R_{s}$$

$$\frac{dR_{r}}{dt}=\phi.I_{r}+\phi.EI_{r}+ \frac{1}{treat_{short}^{duration}}.\left( \delta_{r}^{short} \right).T_{r}^{short}+\frac{1}{treat_{long}^{duration}}. \left( \delta_{r}^{long} \right).T_{r}^{long}+ \frac{1}{treat_{s}^{duration}}.\delta_{bad}. T_{r}^{bad}+ \phi.T_{r}^{short}+\phi.T_{r}^{long}+ \phi.I_{r}^{failed}-{\frac{1}{relapse_{duration}}. \kappa}_{r}.R_{r}- \mu.R_{r}$$

Transmission coefficients for DS and MDR strains are annotated as β_s_ and β_r_, respectively.

Force of infection (FOI): the rate at which susceptible individuals become infected in a population.

The total population will be a combination of all compartments:

$$P=S+L_{s}+EI_{s}+I_{s}+T_{s}+R_{s}+EI_{r}+L_{r}+I_{r}^{bad}+T_{r}^{bad}+ T_{r}^{short}+ T_{r}^{bad}+R_{r}+ I_{r}^{failed}$$

Force of infection for DS strains:

$\lambda_{s}=\beta_{s}.(infI.{EI}_{s}+I_{s}+infT.T_{s})/P$

Force of infection for MDR strains with relatively low transmissibility compared with DS strains ($fit)$

$\lambda_{r}={fit.\beta}_{s}.(infI.{EI}_{r}+I_{r}^{bad}+infT.\left( T_{r}^{short}+T_{r}^{long}+T_{r}^{bad} \right)+I_{r}^{failed})/P$

1. **The Bayesian framework**

Bayesian inference of a model provides a framework for estimating parametric uncertainty in terms of probabilistic distributions and allowing a direct quantification of parameter uncertainty.

Bayes’ theorem states that the best estimate (posterior uncertainty $p(\theta|y$)) for a parameter vector $\theta$ given data y) is given by:

$p\left( \theta| y \right)=\frac{p\left( \theta\right)p(y|\theta)}{p(y)}$

Here, $p\left( \theta\right)$ is the prior information and*,*$\frac{p(y|\theta)}{p(y)}$ is the likelihood ratio. Markov Chain Monte Carlo (MCMC) algorithms were applied to approximate these distributions, which used a sampling scheme to estimate the posterior distribution [16, 17].

## *Prior distribution*

A non-informative uniform prior distribution was chosen to be the prior distribution for all parameter values, to express the absence of prior information about model parameters. The minimum and maximum values were determined initially then narrowed down from the iterative model fitting procedure.

## *Likelihood function*

The likelihood was defined as the product of likelihood terms for each data point. The data arise from WHO Southeast Asia Region TB data from 1990 to 2017 and MDR-TB data from 2020 to 2017 and are linked to the summation of the expected number of TB incident cases, MDR-TB cases, and MDR-TB percentage via a Poisson distribution.

The log-likelihood functions (used as the target in the MCMC algorithm) were:

log-likelihood function of TB cases

${LL}_{TB}=\sum\left( \sum_{t} log\left( \frac{{TB}^{\theta T}exp(-TB)}{\theta T!} \right) \right)$

Where *θT* is the number of TB incident cases in time *t* and *TB* is the expected number of TB incident cases from the model at time *t*.

Log-likelihood function of MDR-TB cases

${LL}_{MDRTB}=\sum\left( \sum_{t} log\left( \frac{{MDRTB}^{\theta M}exp(-MDRTB)}{\theta M!} \right) \right)$

Where *θM* is the number of MDR-TB cases in time *t* and *MDRTB* is the expected number of MDR-TB cases from the model at time *t*.

Log-likelihood function of MDR-TB percentage

${LL}_{PMDRTB}=\sum\left( \sum_{t} log\left( \frac{{PMDRTB}^{\theta P}exp(-PMDRTB)}{\theta P!} \right) \right)$

Where *θP* is the percentage of MDR-TB cases at time *t* and *PMDRTB* is the expected percentage of MDR-TB cases from the model at time *t*.

## *Posterior distribution estimation*

We used a Differential Evolution Markov Chain Monte Carlo (MCMCzs, or DE-MCzs) method to estimate the posterior distributions. We used the Markov Chain methods of sampling proposed by Ter Braak and Vrugt [18], which have previously been used for numerical problems, and implemented them using the Bayesian Tools R package. Differential Evolution Markov Chain (DE-MC) is an adaptive MCMC algorithm, in which multiple chains are run in parallel and presented. The DE-MCzs method combines characteristics of conventional MCMC methods with the ideas of differential evolution optimization algorithms, by making use of the full joint density function and (independent) proposal distributions for each of the variables, including effective contact rate parameters. These samples were accepted probabilistically based on the acceptance probability. Uniform distributions are centered at the current state of the chain. This proposal distribution randomly perturbs the current state of the chain and then either accepts or rejects the perturbed value. Two chains were run in parallel on three cores, each consisting of 200,000 iterations, with a burn-in period of 10,000 iterations, to achieve a target acceptance rate of 0.327. The display of the two-split chains when using the Bayesian package was 6 lines of (200,000-10,000)/3 = 63,333.33, as shown in **S2 Fig**.

**S1 Table**. WHO Global TB Report data of median estimates used for the model fitting.

| **Year** | **TB incidence per 100,000 per year**  **(median estimate)** | **MDR-TB precentage among new TB cases**  **(median estimate)** |
| --- | --- | --- |
| 1990 | 302.5 |  |
| 1991 | 296.2 |  |
| 1992 | 290.7 |  |
| 1993 | 285.6 |  |
| 1994 | 281.1 |  |
| 1995 | 276.7 |  |
| 1996 | 273 |  |
| 1997 | 270 |  |
| 1998 | 267.3 |  |
| 1999 | 264.7 |  |
| 2000 | 262 |  |
| 2001 | 259.3 |  |
| 2002 | 256.1 |  |
| 2003 | 252.7 |  |
| 2004 | 248.8 |  |
| 2005 | 244.8 |  |
| 2006 | 240.8 |  |
| 2007 | 236.7 |  |
| 2008 | 232.6 |  |
| 2009 | 229 |  |
| 2010 | 225.9 | 2.1% |
| 2011 | 223.4 | 2.1% |
| 2012 | 221.9 | 2.2% |
| 2013 | 220.4 | 2.2% |
| 2014 | 220.3 | 2.2% |
| 2015 | 246 | 2.6% |
| 2016 | 240 | 2.8% |
| 2017 | 226 | 2.7% |

**
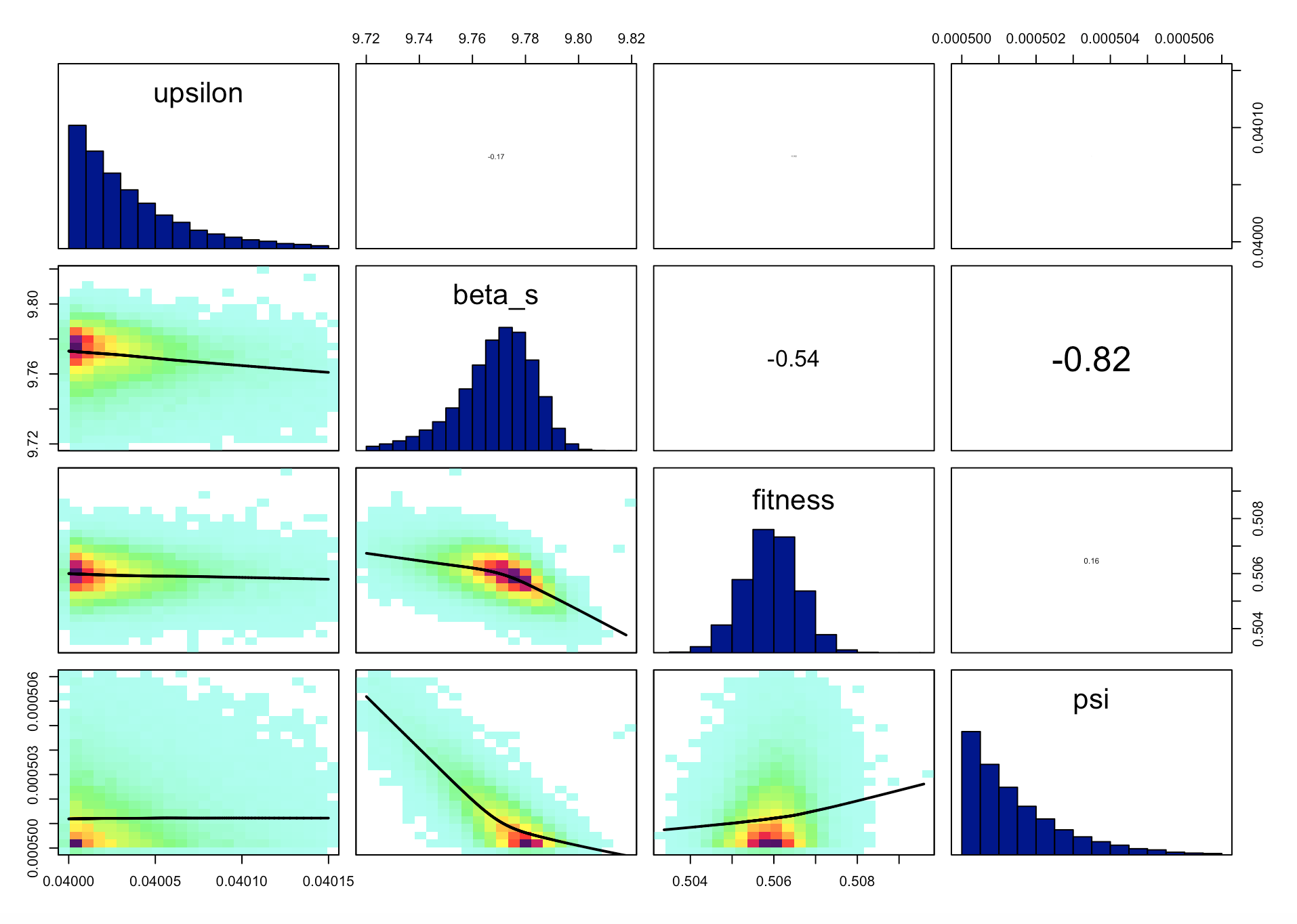
**

**S1 Fig**. Correlation plot for the parameters fitted (upsilon = proportion of rapid progression to active TB; beta_s = effective contact rate for drug-sensitive TB; fitness = relative transmissibility of MDR-TB compared with that of drug-sensitive TB; psi = reactivation rate from latent to active TB).

**
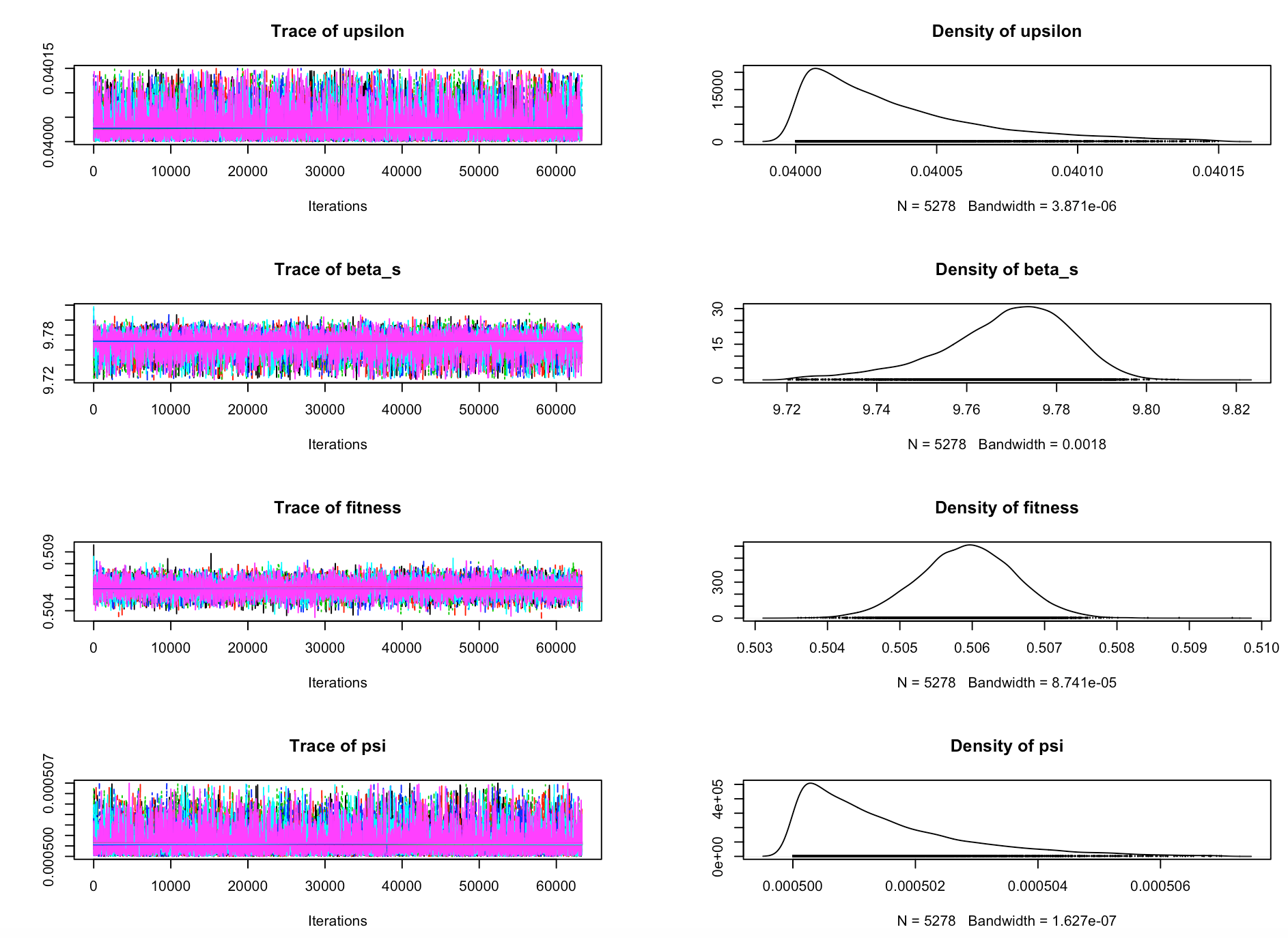
**

**S2 Fig.** Posterior distributions from the TB model, with each row corresponding to a separate parameter; the left-hand column contains traces with 6 color chains (dashed lines: actual traces, solid lines: trends) and the right-hand column contains the posterior distribution, corresponding to each parameter.

**
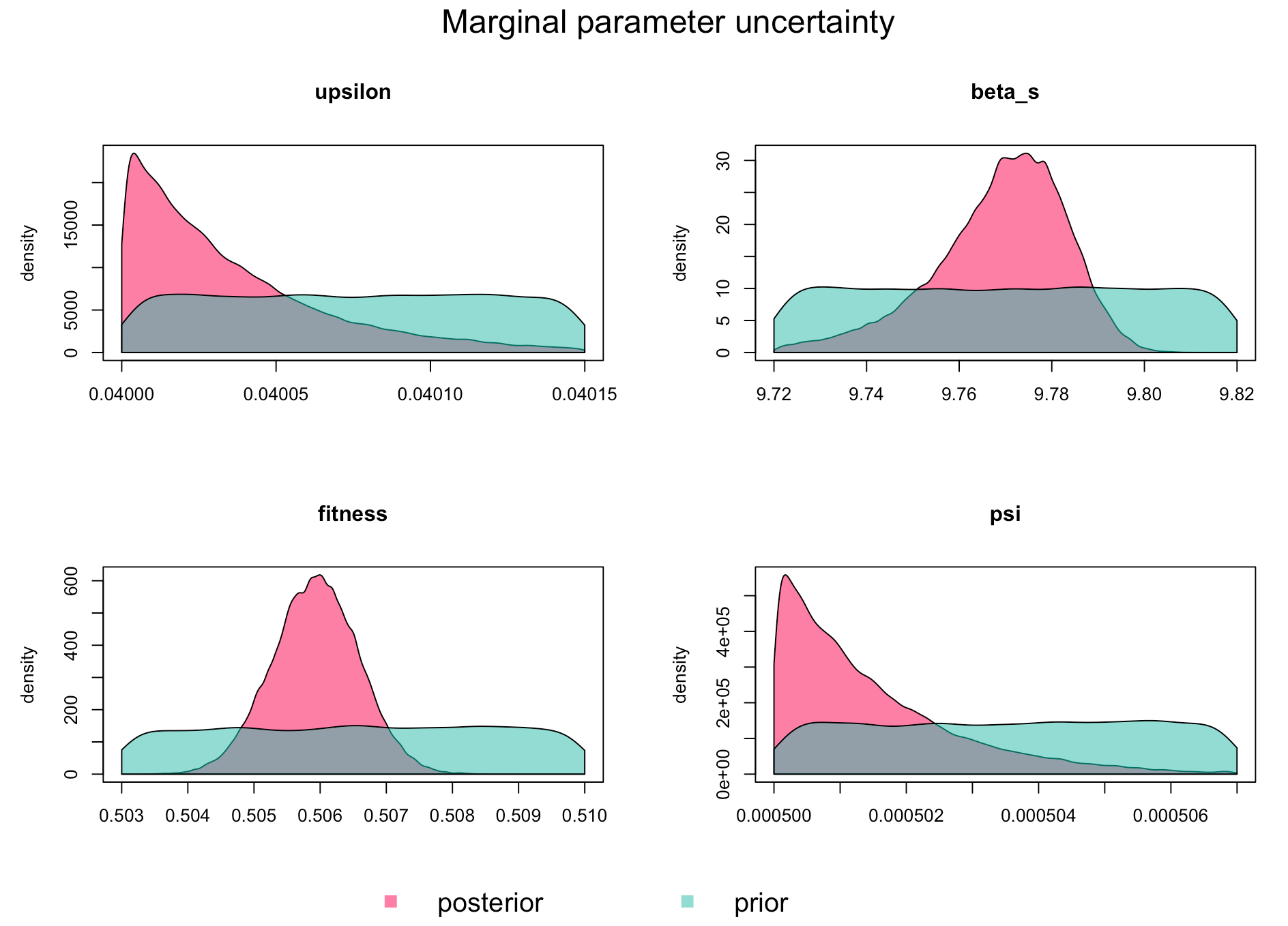
**

**S3 Fig.** Marginal parameter uncertainty.

**S1 Appendix – References**

1. Diel R, Loddenkemper R, Niemann S, Meywald-Walter K, Nienhaus A. Negative and positive predictive value of a whole-blood interferon-gamma release assay for developing active tuberculosis: an update. Am J Respir Crit Care Med. 2011;183(1):88-95.

2. Dye C. The potential impact of new diagnostic tests on tuberculosis epidemics. Indian J Med Res. 2012;135:737-44.

3. Hickson RI, Mercer GN, Lokuge KM. A metapopulation model of tuberculosis transmission with a case study from high to low burden areas. PLoS One. 2012;7(4):e34411.

4. Crampin AC, Mwaungulu JN, Mwaungulu FD, Mwafulirwa DT, Munthali K, Floyd S, et al. Recurrent TB: relapse or reinfection? The effect of HIV in a general population cohort in Malawi. AIDS. 2010;24(3):417-26.

5. Lambert ML, Hasker E, Van Deun A, Roberfroid D, Boelaert M, Van der Stuyft P. Recurrence in tuberculosis: relapse or reinfection? Lancet Infect Dis. 2003;3(5):282-7.

6. Rodrigues P, Gomes MG, Rebelo C. Drug resistance in tuberculosis--a reinfection model. Theor Popul Biol. 2007;71(2):196-212.

7. Sergeev R, Colijn C, Cohen T. Models to understand the population-level impact of mixed strain M. tuberculosis infections. J Theor Biol. 2011;280:88-100.

8. Vynnycky E, Fine PE. The annual risk of infection with Mycobacterium tuberculosis in England and Wales since 1901. Int J Tuberc Lung Dis. 1997;1(5):389-96.

9. Kendall EA, Fofana MO, Dowdy DW. Burden of transmitted multidrug resistance in epidemics of tuberculosis: a transmission modelling analysis. Lancet Respir Med. 2015;3(12):963-72.

10. World Health Organization. Global tuberculosis report 2018. Geneva; 2018.

11. Vynnycky E, Fine PE. Interpreting the decline in tuberculosis: the role of secular trends in effective contact. Int J Epidemiol. 1999;28(2):327-34.

12. Kendall EA, Fojo AT, Dowdy DW. Expected effects of adopting a 9 month regimen for multidrug-resistant tuberculosis: a population modelling analysis. The Lancet Respiratory medicine. 2017;5(3):191-9.

13. Blower SM, McLean AR, Porco TC, Small PM, Hopewell PC, Sanchez MA, et al. The intrinsic transmission dynamics of tuberculosis epidemics. Nat Med. 1995;1(8):815-21.

14. Fox GJ, Barry SE, Britton WJ, Marks GB. Contact investigation for tuberculosis: a systematic review and meta-analysis. The European respiratory journal : official journal of the European Society for Clinical Respiratory Physiology. 2013;41(1):140-56.

15. Horsburgh CR, Jr., O'Donnell M, Chamblee S, Moreland JL, Johnson J, Marsh BJ, et al. Revisiting rates of reactivation tuberculosis: a population-based approach. Am J Respir Crit Care Med. 2010;182(3):420-5.

16. Augustynczik ALD, Hartig F, Minunno F, Kahle H-P, Diaconu D, Hanewinkel M, et al. Productivity of Fagus sylvatica under climate change – A Bayesian analysis of risk and uncertainty using the model 3-PG. Forest Ecology and Management. 2017;401:192-206.

17. Hartig F, Minunno F, Paul S. BayesianTools: General-Purpose MCMC and SMC Samplers and Tools for Bayesian Statistics. R package version 0.1.6. 2019 [Available from: <https://github.com/florianhartig/BayesianTools>.

18. Ter Braak CJ, Vrugt, J.A. Differential evolution Markov chain with snooker updater and fewer chains. Stat Comput 2008;18:435–46.
